# Correction: Liu et al. Rational Design of Nitrogen-Doped Carbon Dots for Inhibiting β-Amyloid Aggregation. *Molecules* 2023, *28*, 1451

**DOI:** 10.3390/molecules29102253

**Published:** 2024-05-11

**Authors:** Hong Liu, Huazhang Guo, Yibin Fang, Liang Wang, Peng Li

**Affiliations:** 1 Department of Neurovascular, Shanghai Fourth People’s Hospital, Tongji University School of Medicine, Shanghai 200434, China; 2 Department of Neurology, Shanghai East Hospital, Tongji University School of Medicine, Shanghai 200120, China; 3 Institute of Nanochemistry and Nanobiology, School of Environmental and Chemical Engineering, Shanghai University, Shanghai 200444, China

(1) Error in Figure

In the original publication [1], there was a mistake in “Figure 3f” as published. “In Figure 3f, the high-resolution O1s spectrum of N-CDs was replaced by C1s spectrum.” The corrected “Figure 3f” appears below.

(2) Text Correction

There was an error in the original publication. “In Figure 3f, the XPS O1s spectrum was distributed to C-O, C=O, and O=C-NH at 530.88, 532.30, and 533.82 eV, respectively.”

A correction has been made to “In Figure 3f, the XPS O1s spectrum was distributed to C=O, O=C-NH, and C-O, at 530.88, 532.30, and 533.82 eV, respectively.”

The authors state that the scientific conclusions are unaffected. This correction was approved by the Academic Editor. The original publication has also been updated.

## Figures and Tables

**Figure 3:**
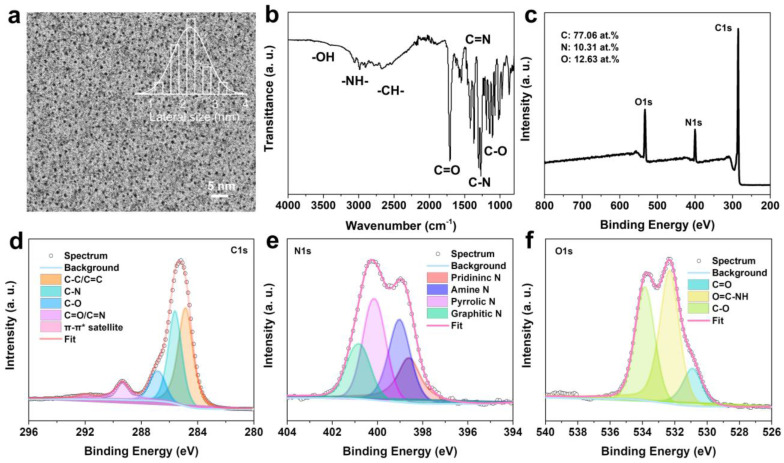
(**a**) TEM image and its corresponding lateral size distribution, (**b**) FT-IR spectrum, (**c**) survey XPS spectrum, (**d**) high-resolution C1s spectrum, (**e**) high-resolution N1s spectrum, and (**f**) high-resolution O1s spectrum of N-CDs.
