# Supplementary material for: Rational Design of Nitrogen-Doped Carbon Dots for Inhibiting β-Amyloid Aggregation
Source: Molecules. 2023 Feb 2;28(3):1451. doi: 10.3390/molecules28031451 (PMC9919344; doi:10.3390/molecules28031451)
Supplement: Supplementary file 1 [file molecules-28-01451-s001.zip › molecules-2153938-supplementary.pdf]

Supporting Information

Rational Design of Nitrogen-Doped Carbon Dots for Inhibiting β-Amyloid Aggregation

Hong Liu [1,†], Huazhang Guo [2,†], Yibin Fang [3,*], Liang Wang [2,*] and Peng Li [3,*]

[1] Department of Neurology, Shanghai East Hospital, Tongji University School of Medicine, Shanghai 200120, China
[2] Institute of Nanochemistry and Nanobiology, School of Environmental and Chemical Engineering, Shanghai University, Shanghai 200444, China
[3] Department of Neurovascular, Shanghai Fourth People's Hospital, Tongji University School of Medicine, Shanghai 200434, China
* Correspondence: 13585831041@163.com (Y.F.); wangl@shu.edu.cn (L.W.); 15921196270@126.com (P.L.)
† These authors contributed equally to this work.

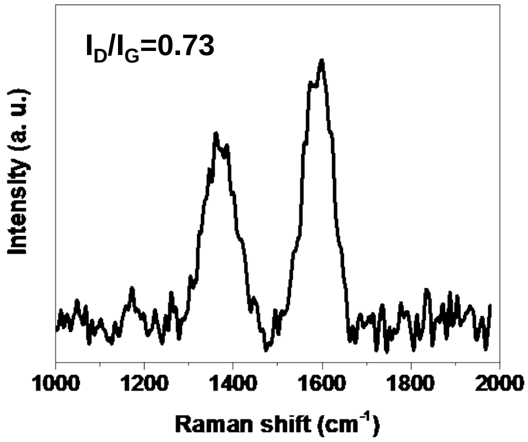

Figure S1. Raman spectrum of N-CDs.
